# Arboviral infections and pregnancy: An overlooked public health challenge

**DOI:** 10.1016/j.nmni.2025.101587

**Published:** 2025-04-15

**Authors:** Ibrahim Nagmeldin Hassan

**Affiliations:** University of Khartoum, Khartoum, Sudan

Dear Editor,

Arboviral infections have emerged as a significant global health challenge, yet their impact during pregnancy remains underexplored, particularly in regions where these infections are endemic. Arboviruses such as Zika, Dengue, Chikungunya, Yellow Fever, and Oropouche viruses disproportionately affect low- and middle-income countries (LMICs), where 85 % of arboviral disease cases are reported annually [1]. Despite this, there is limited research on the burden of these infections in pregnant women and their implications for maternal and fetal health.

Pregnancy alters the immune system, increasing susceptibility to infections and exacerbating their potential complications. Adverse maternal outcomes, including severe febrile illness, neurological symptoms, and life-threatening hemorrhagic manifestations (notably in Dengue, which infects 390 million people annually) [2], have been documented. Of these infections, approximately 96 million cases are clinically apparent, including thousands in pregnant women. In addition, vertical transmission poses significant risks to fetal health. For example, the Zika virus epidemic of 2015–2016 highlighted its devastating consequences, with congenital Zika syndrome affecting 10 % of infants born to infected mothers [3]. The broader implications of arboviruses like Dengue and Oropouche during pregnancy remain poorly understood, although adverse outcomes such as miscarriage, stillbirth, preterm birth, and low birth weight have been suggested in several reports [4].

One of the primary barriers to addressing arboviral infections during pregnancy is the lack of accurate diagnostic capabilities. Arboviruses often present with nonspecific symptoms—fever, rash, and arthralgia—that overlap with other febrile illnesses, such as malaria, typhoid, and bacterial infections, making clinical diagnosis challenging. Moreover, laboratory confirmation is limited in endemic regions. For instance, molecular diagnostic tools such as reverse transcription-polymerase chain reaction (RT-PCR) remain scarce in LMICs, where 2.5 billion people are at risk of Dengue infection alone [5]. Serological tests often yield inconclusive results due to cross-reactivity among related arboviruses, such as Dengue and Zika. These diagnostic limitations contribute to underreporting and impede timely treatment, particularly for pregnant women, whose care requires greater precision and urgency.

Another challenge lies in the insufficient data on the epidemiology of arboviral infections during pregnancy. While Zika virus research surged following its association with microcephaly and other neurological anomalies in over 4800 reported cases during the 2015–2016 epidemic, other arboviruses remain neglected. For instance, Dengue, the most common arbovirus globally, affects pregnant women in endemic areas, yet its maternal and fetal outcomes remain poorly quantified. Studies suggest that pregnant women with Dengue are at a higher risk of preterm delivery and postpartum hemorrhage, with mortality rates in some outbreaks exceeding 1 % [2]. Similarly, the Oropouche virus, which has caused over 500,000 cases of febrile illness in South America, remains vastly understudied in pregnancy, despite evidence of its neurotropic potential and the likelihood of vertical transmission.

The public health response to arboviral infections in pregnancy is further hampered by healthcare inequities. Endemic regions, which bear the brunt of these infections, often lack sufficient healthcare infrastructure. For instance, only 45 % of pregnant women in LMICs receive adequate prenatal care, limiting opportunities for early detection and intervention [4]. Vector control measures, such as insecticide-treated nets and indoor residual spraying, remain insufficiently implemented, leaving millions of pregnant women at risk of arboviral infections.

To address these gaps, several strategies are urgently needed. First, affordable, rapid diagnostic tests must be developed and distributed to endemic regions. The availability of point-of-care tests capable of differentiating arboviruses from other febrile illnesses could significantly enhance early detection and management, particularly for pregnant women. Second, surveillance systems must be strengthened to monitor arboviral infections in pregnancy and their outcomes. For instance, integrating arboviral surveillance into existing maternal health programs could provide critical data, particularly for under-researched viruses like Chikungunya and Oropouche.

Third, research funding must prioritize the study of arboviral infections during pregnancy. Investigations into the pathophysiology of arbovirus transmission during pregnancy, as well as the safety and efficacy of vaccines, are essential. While vaccines for Yellow Fever and Dengue exist, they remain underutilized in pregnant women due to insufficient safety data. The development of vaccines for other arboviruses, such as Zika and Chikungunya, should also consider pregnant women as a key target population.

Finally, public health interventions must address systemic inequities. Community-based vector control initiatives, improved access to prenatal care, and education campaigns targeting pregnant women can reduce the burden of arboviral infections. For example, scaling up the use of insecticide-treated nets, which have been shown to reduce mosquito-borne infections by 50 % [5], could substantially lower transmission rates among pregnant women.

Arboviral infections during pregnancy represent an overlooked public health challenge with far-reaching consequences for maternal and fetal health. Addressing this issue requires a coordinated global response that combines improved diagnostics, strengthened surveillance, targeted research, and equitable healthcare interventions. By prioritizing the needs of pregnant women in arbovirus-endemic regions, we can mitigate the impact of these infections and improve health outcomes for mothers and their children.

## Funding

No funds, grants, or other support was received.

## Availability of data and materials

Not applicable.

## Ethics approval and consent to participate

Not applicable.

## Consent for publication

Not applicable.

## Funding

No funding was received for this work.

## Declaration of competing interest

The authors declare that they have no known competing financial interests or personal relationships that could have appeared to influence the work reported in this paper.
